# Machine Learning na Medicina: Revisão e Aplicabilidade

**DOI:** 10.36660/abc.20200596

**Published:** 2022-01-01

**Authors:** Gabriela Miana de Mattos Paixão, Bruno Campos Santos, Rodrigo Martins de Araujo, Manoel Horta Ribeiro, Jermana Lopes de Moraes, Antonio L. Ribeiro

**Affiliations:** 1 Universidade Federal de Minas Gerais Belo Horizonte MG Brasil Universidade Federal de Minas Gerais, Belo Horizonte, MG – Brasil; 2 Universidade Federal do Ceará Sobral CE Brasil Universidade Federal do Ceará, Sobral, CE – Brasil

**Keywords:** Aprendizado de Máquina, Medicina, Cardiologia

## Introdução

O aprendizado de máquina, ou *machine learning* (ML), é um ramo da inteligência artificial (IA) que explora o estudo e a construção de algoritmos computacionais a partir do aprendizado por dados,^1,2^ ao invés de instruções pré–programadas.^3^ O objetivo principal de um modelo de ML é construir um sistema de computador que aprenda com um banco de dados pré–definido e gere, ao final, um modelo de predição, classificação ou detecção.

A aplicação de ML na prática é voltada principalmente para o manuseio de bases de dados consolidadas com informações heterogêneas, para as quais há uma limitação do uso das técnicas de estatística convencionais.^4,5^ Os algoritmos de ML já estão difundidos em diversas áreas, como sistemas bancários para detecção de fraudes, mecanismos de busca na internet, sistemas de vigilância em vídeo, segurança de dados, logística de empresas, robótica e, na medicina, para diagnóstico e prognóstico.^6^ Com a digitalização dos prontuários médicos, exames laboratoriais e de imagem, houve um crescimento dos bancos de dados. Esses são fontes para a aplicação de técnicas de ML, visando a prevenção, diagnóstico precoce e o tratamento das doenças.

Este artigo de revisão aborda uma introdução sobre ML dividida em: definição, modelos de aprendizagem e uma revisão sistemática de artigos sobre a sua aplicabilidade na medicina e, principalmente, na cardiologia. O objetivo é apresentar ML para médicos e profissionais de saúde como uma ferramenta de auxílio para a prática clínica.

Para a estruturação deste artigo de revisão foram pesquisadas duas bases de dados: PubMed (NCBI) e Medline, os seguintes descritores na língua inglesa: “*machine learning*”, “*artificial intelligence*”, “*unsupervised learning*”, “*supervised learning*”, “*neural networks*” e “*cardiology*”. Foram incluídos: estudos prospectivos e retrospectivos, excluídos: casos clínicos e resumos apresentados em congressos (não publicados sob a forma de artigo). A elegibilidade de cada estudo foi avaliada por dois investigadores. As opiniões divergentes relativamente à relevância dos artigos foram abordadas por consenso entre os autores.

### Machine learning

O aprendizado de máquina é um subcampo da ciência da computação que busca uma interseção de técnicas matemáticas e estatísticas com algoritmos computacionais.^3,7^ ML utiliza algoritmos com o conceito de IA e é aplicada em determinadas situações em que se busca padrões em um conjunto de variáveis com o intuito de prever um resultado específico de interesse.^8,9^

A maioria das técnicas convencionais usadas em sistemas computacionais aplicados à medicina empregam o conceito de algoritmos baseados em regras, chamados de “sistemas especialistas”. Assim, o desenvolvedor codifica os conhecimentos médicos sobre um determinado assunto para esses sistemas, utilizando regras já conhecidas. Já as técnicas de ML manuseiam um grande número de variáveis, buscando uma variedade de novas combinações que possam prever um resultado com confiabilidade, muitas vezes, em uma grande quantidade de dados, tais como *big data*.^7^

Em 2001, Doug Laney definiu um modelo de “3 Vs” para conceituar o termo *big data*: grande volume, alta velocidade e alta variedade de informações exigem novas técnicas de processamento de forma a permitir descobertas e otimizar processos.^10^ O termo *big data* pode ser tanto um conjunto de dados de tamanho enorme, que nenhuma das ferramentas tradicionais de gerenciamento de dados é capaz de armazená–los ou processá–los com eficiência, como também pode se referir a um tipo de tecnologia (como instalações de armazenamento, ferramentas e processos).^11^

**Figura 1:**
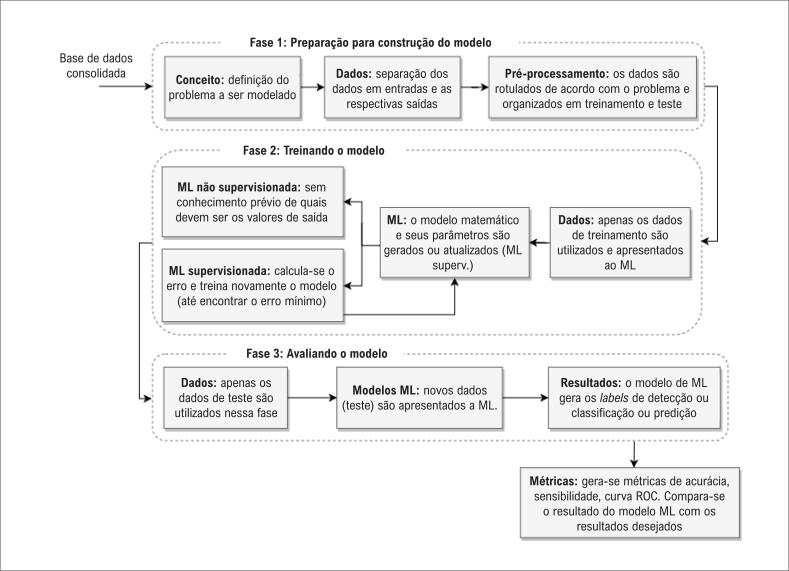
*Fases para o desenvolvimento de algoritmos de machine learning*.^*15*^

O processo de desenvolvimento de um algoritmo de ML é dividido em três fases: pré–processamento, treinamento e avaliação do modelo (Figura 1). A primeira fase consiste em organizar o banco de dados, definir a pergunta de pesquisa e dividir os dados em treinamento e teste. No treinamento, o aprendizado pode ocorrer de forma supervisionada ou não supervisionada.^12–15^ O aprendizado supervisionado é baseado no treinamento de uma amostra de dados com a classificação correta já atribuída, enquanto o não supervisionado se refere à capacidade de aprender e organizar informações sem a atribuição da classificação correta.^14^ Na fase de avaliação, o modelo é comparado com os dados de teste e os resultados são gerados. Portanto, os algoritmos de ML aprendem através de repetidas observações e estabelecem um padrão de mapeamento com o intuito de rotular os dados e criar um modelo que generaliza as informações, de modo que novos dados (jamais analisados pelo algoritmo) possam ser rotulados com precisão e confiabilidade.^15^

É importante salientar que o processo de desenvolvimento de um algoritmo de ML deve ser realizado com uma base de dados consolidada e validada, pois modelos de ML desenvolvidos com dados não consolidados podem gerar resultados enganosos.^5^

### *Machine learning* supervisionada e não supervisionada

A principal diferença entre os modelos de aprendizagem supervisionado e não supervisionado está no algoritmo de treinamento. No aprendizado não supervisionado, o modelo de ML extrai as características dos dados e constrói uma representação sem o conhecimento prévio dos rótulos de cada dado, ou seja, identifica o padrão das informações de classe heuristicamente. Essa falta de supervisão para o algoritmo pode ser vantajosa, pois permite que o algoritmo analise os padrões que não foram considerados anteriormente.^12–14^

No aprendizado supervisionado, o modelo do ML tem o conhecimento do rótulo dos dados, ou seja, as amostras estão corretamente classificadas. O treinamento é baseado na comparação entre o resultado obtido do modelo e o rótulo previamente classificado. Esse processo é repetido até se obter um erro mínimo.^14^

A Tabela 1 resume as principais características de cada tipo de modelo de aprendizado, bem como suas vantagens e desvantagens e aplicabilidade prática.

**Tabela 1:** Comparação entre processo de aprendizagem supervisionado e não supervisionado

	Aprendizado supervisionado	Aprendizado não supervisionado
Definição	Algoritmos que aprendem relações entre atributos de entrada e de saída a partir de conjunto de exemplos rotulados	Algoritmos que buscam encontrar padrões em agrupamentos de dados com características semelhantes, em busca de categorias e desfechos ainda não identificados ou não informados
Vantagens	Análise de múltiplos parâmetros. Solução rápida e automática para questões de grande escala e elevada acurácia	Menor interferência humana na análise dos dados. Excelente excelente para fontes de dados multimodais ou multidimensionais. Permite identificação de novos desfechos
Desvantagens	Necessidade dos dados serem rotulados, o que para grandes volumes de dados pode ser impraticável. Tendência ao sobreajuste dos dados	Custo elevado e técnicas complexas. Necessita grande quantidade de dados para elaboração do algoritmo. Interpretação dos resultados pode ser desafiadora
Principais tarefas	Regressão, classificação, modelo prognóstico e análise de sobrevivência	Redução da dimensionalidade do problema e agrupamento
Exemplos de algoritmos	Regressão logística, árvores de decisão, *random forests* e redes neurais artificiais	Análise das componentes principais, agrupamento hierárquico, *autoencoders*, análise linear de discriminantes

### Técnicas de *machine learning*

Diversas técnicas de ML têm sido aplicadas como forma de sistemas de diagnóstico auxiliado por computador, tais como: redes neurais artificiais (RNAs), regressão logística, árvore de decisão, *random forests*, rede bayesiana, *deep learning, support vector machine* (SVM), entre outros.^16–21^ Algumas técnicas utilizam modelos matemáticos por meio dos dados para aprendizagem e/ou organização das informações.^12^ Outras utilizam representações matemáticas com alto grau de abstração (modelos matemáticos complexos). Neste caso, não é possível decifrar ou interpretar os métodos utilizados para obtenção dos resultados de predição, detecção ou classificação, de modo que tais modelos de ML são chamados de “caixa preta”.^22^

**Figura 2:**
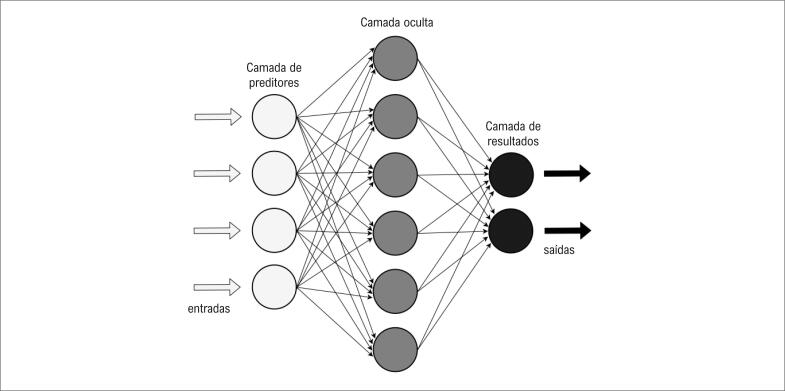
*Estrutura do funcionamento de uma rede neural artificial*.^*19*^

Uma RNA é um modelo computacional e matemático desenvolvido para funcionar como o cérebro humano. Uma RNA possui diversos elementos de interconexões (camada de preditores, camada oculta e camada de resultados). A relação entre essas camadas é inspirada nas conexões sinápticas entre os neurônios (Figura 2).^12,15,23^

Uma RNA “aprende” através dessas conexões entre as camadas (preditores, oculta e resultados) e os pesos associados a cada camada. Sendo assim, um dado de entrada é apresentado na camada de preditores, sendo esse enviado camada a camada. O processamento matemático ocorre no envio de dados de uma camada a outra e os pesos dessas conexões são atualizados de acordo com o erro da camada de resultados, ou seja, a relação do resultado esperado e o resultado obtido. Esse processo é repetido até o valor do erro ser mínimo ou um valor especificado de interações.^12,23,24^

*Deep learning* difere o seu aprendizado das técnicas mais tradicionais de ML, pois processa modelos computacionais mais robustos e com múltiplas camadas de processamento baseadas em RNAs. Sendo assim, a técnica de *deep learning* funciona semelhante a uma RNA, mas possuindo um número maior de camadas ocultas e, consequentemente mais conexões sinápticas. Cada camada reproduz uma representação dos dados oriundos da camada anterior e seu algoritmo de aprendizado pode ser tanto supervisionado como não supervisionado.^25,26^

Com o grande volume e a complexidade dos dados que envolvem trabalhar com *big data*, o algoritmo do *autoencoder* é um tipo de RNA que reduz a dimensionalidade dos dados. Para isso, esse algoritmo utiliza modelos matemáticos com alto grau de abstração para gerar um novo conjunto de dados reduzidos em dimensionalidade com representação o mais próxima possível dos dados de entrada. A diferença fundamental entre a RNA e o *autoencoder* é que o último utiliza em sua fase de treinamento dados não–rotulados.^27^

O algoritmo da árvore de decisão é o mais utilizado quando o conjunto de dados é relativamente pequeno e é desenvolvido com uma série de perguntas de sim/não para classificar os dados em categorias. Esse algoritmo utiliza um modelo estatístico para classificação ou predição de dados. Cada pergunta se divide em possíveis resultados e esses se ramificam em outras possibilidades; isso se repete até um desfecho final.^16^ As principais vantagens deste algoritmo são sua simplicidade e interpretação intuitiva.^28^

*Random forests* são uma ampliação do algoritmo da árvore de decisão, sendo bastante utilizado para resolução de problemas de classificação e regressão. As árvores de decisão são combinadas e cada uma é treinada independentemente. Suas principais características são: teoria simples, velocidade rápida na análise dos dados, estabilidade com a presença de excesso de ruído e mecanismo de compensação automática em amostras tendenciosas dos dados.^29^

A rede bayesiana é outra técnica muito aplicada à medicina. Métodos estatísticos bayesianos com uma fundamentação teórica que crenças subjetivas coerentes a especialistas de uma determinada área podem ser expressas em uma estrutura probabilística.^17^

O SVM é um método de ML com aprendizado supervisionado, amplamente utilizado em bioinformática. Este algoritmo utiliza a ideia de minimização do erro e trabalha com teoria estatística do aprendizado e da otimização. Além da classificação binária, o SVM pode ser usado na regressão de dados contínuos, chamado de regressão do vetor de suporte. Os resultados obtidos com o uso do SVM são comparáveis aos de RNAs, apresentando processo de treinamento fácil e trabalhando com alta dimensionalidade de dados. Portanto, esse encontra um compromisso entre menor complexidade e erro.^30,31^

Dessa forma, cada algoritmo utiliza técnicas distintas de como aprender com observações e como realizar um mapeamento do conjunto de preditores para o resultado final. Esse deve generalizar as informações, de modo que uma tarefa possa ser executada corretamente com entradas novas, não analisadas anteriormente pelo modelo.^14^

### *Machine learning* na medicina

**Figura 3:**
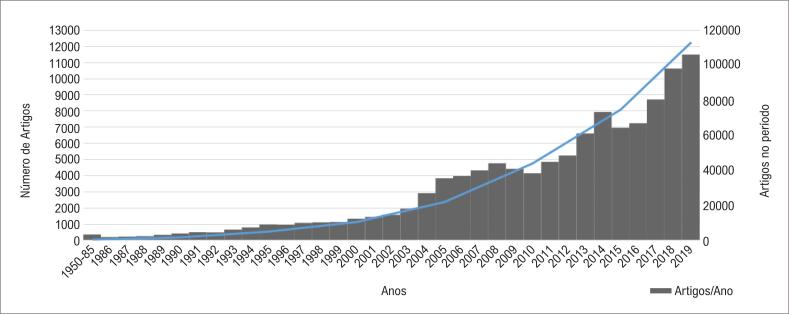
*Número de artigos por ano e acumulado durante o período de 1951 a 2019 no PubMed e Medline*.

Desde o século passado, os pesquisadores exploram as diversas aplicações das técnicas de ML em todos os campos da medicina.^32^ A pesquisa médica envolvendo ML tem crescido exponencialmente ao longo das últimas décadas. Os dados do PubMed (NCBI) e Medline, envolvendo os descritores “*machine learning*”, “*artificial intelligence*”, “*unsupervised learning*”, “*supervised learning*” e “*neural networks*”, revelou 113.127 artigos publicados entre 1951 e 2019 (Figura 3). Ao acrescentar–se o descritor “*cardiology*” como condição obrigatória na pesquisa dos demais termos, 888 trabalhos retornam com distribuição semelhante à anterior, entre os anos de 1986 e 2019.

A capacidade dos algoritmos de ML de reconhecer padrões e predizer diagnóstico tem sido amplamente aplicada às diversas áreas de atenção à saúde.^33–36^ Na dermatologia, uma RNA foi capaz de diferenciar lesões dermatológicas em benignas versus malignas, a partir de mais de 129.000 casos, com resultados similares a um comitê de 21 dermatologistas.^35^ No campo da psiquiatria, o estudo com técnicas de ML reduziu o número de critérios diagnósticos de 29 para 8 com 100% de acurácia em 612 pacientes com diagnóstico firmado de transtorno do espectro autista.^36^

A adição de tecnologias móveis, tais como: *smartphone* e *smartwatches*, aplicadas à área da saúde acrescentou mais uma dimensão ao ML, permitindo a leitura de grandes quantidades de dados pessoais em algoritmos de aprendizado.^37^ Dentro de sistemas de *feedback*, a tecnologia móvel consegue ser um dispositivo biométrico (por exemplo, medir os níveis de glicose no sangue) com capacidade de direcionamento para intervenções clínicas em tempo real, baseadas em algoritmos que atualizam continuamente as informações pessoais do paciente.^38^ A tecnologia pode simplificar os processos diagnósticos e facilitar a prática clínica.

### *Machine learning* na cardiologia

O avanço na capacidade computacional nas últimas décadas impactou especialmente o campo da detecção e predição de doenças cardiovasculares por meio da interpretação de dados, como: estudos dos prontuários médicos, exames de imagem, banco de dados biológicos e genômicos e de avaliação molecular.^32^ A cardiologia é uma das áreas de maior impacto na produção científica usando técnicas de ML (Tabela 2). Desde a predição de eventos cardiovasculares^39^ à melhoria dos diagnósticos eletrocardiográficos,^40,41^ a IA tem sido ferramenta importante para a pesquisa cientifica.

**Tabela 2:** Artigos com o uso das técnicas *machine learning* na cardiologia

Artigo	Principais resultados
Can machine-learning improve cardiovascular risk prediction using routine clinical data?^38^	O algoritmo foi capaz de predizer 4998 de 7404 casos positivos (sensibilidade 67,5%, VPP 18,4%) e 53458 de 75585 casos negativos (especificidade 70,7% e VPN 95,7%), com ganho de 355 pacientes (+7,6%) que desenvolveram doenças cardiovasculares em relação ao método tradicional.
Deep neural networks can predict mortality from 12-lead electrocardiogram voltage data^43^	Por meio da análise isolada do ECG por algoritmo de ML, foi possível predizer mortalidade por todas as causas em um ano com AUC = 0,84 e p < 0,05.
Phenomapping for the Identification of Hypertensive Patients with the Myocardial Substrate for Heart Failure with Preserved Ejection Fraction^56^	Um grupo de 1273 pacientes hipertensos foi avaliado por meio de técnicas de ML, utilizando dados clínicos, laboratoriais e ecocardiográficos. Foi possível identificar um grupo de pacientes com maior risco de desenvolver insuficiência cardíaca de fração preservada que, provavelmente, devem ser beneficiar de tratamento clínico mais intensivo.
Cognitive Machine-Learning Algorithm for Cardiac Imaging: A Pilot Study for Differentiating Constrictive Pericarditis From Restrictive Cardiomyopathy^57^	Utilizaram técnicas de ML para diferenciar pericardite constritiva de cardiomiopatia restritiva com uma curva ROC de 96,2% e acurácia superior a 90%.
Structured learning algorithm for detection of nonobstructive and obstructive coronary plaque lesions from computed tomography angiography^58^	O algoritmo de ML foi capaz de detectar lesões coronarianas superiores ou iguais a 25% com uma sensibilidade 93%, especificidade 95% e acurácia de 94% em 42 angiografias coronárias.
A deep neural network learning algorithm outperforms a conventional algorithm for emergency department electrocardiogram interpretation^54^	A análise automática pelo método de ML para a leitura de ECG em um departamento de emergência obteve sensibilidade (88,7% versus 92,0%, p < 0,086), especificidade (94% versus 84,7%, p < 0,0001), VPP (88,2% versus 75,4%, p < 0,0001) e acurácia (92,2% versus 87,2%, p < 0,0001) em relação ao método automático convencional.
Automatic Diagnosis of the Short-Duration 12-Lead ECG using a Deep Neural Network: the CODE Study^53^	Uma rede neural treinada foi capaz de detectar 6 classes de anormalidades eletrocardiográficas com especificidade superior a 99% e performance superior a 80%, comparada com residentes de cardiologia do último ano.
An artificial intelligence-enabled ECG algorithm for the identification of patients with atrial fibrillation during sinus rhythm: a retrospective analysis of outcome prediction^55^	Um software de ML foi capaz de detectar pacientes portadores de fibrilação atrial, a partir de ECG em ritmo sinusal com uma sensibilidade de 79%, especificidade 79,5% e acurácia de 79,4%.

*ECG: eletrocardiograma; ML: machine learning; VPN: valor preditivo negativo; VPP: valor preditivo positivo*.

### Prognóstico

Diversos escores de risco cardiovasculares foram desenvolvidos no intuito de predizer eventos cardiovasculares e identificar os indivíduos com maior risco cardíaco para a prevenção primária.^42^ No entanto, a despeito de todo o avanço propedêutico e terapêutico na cardiologia, ainda há uma população em risco não identificada pelos métodos tradicionais.^43^ O reconhecimento de potenciais fatores de risco não tradicionais é desejável e o uso de novas tecnologias, como a IA, torna–se método promissor nessa busca.

A predição de mortalidade por todas as causas no período de um ano, a partir da análise isolada do eletrocardiograma (ECG), apresentou resultados promissores (AUROC 0,87; p < 0,05).^44^ É interessante ressaltar que uma análise cega destes ECG feita por três cardiologistas sugere que os padrões encontrados para predizer mortalidade pelo ML não são aparentemente visíveis pela avaliação médica convencional.^44^

Em estudo com 2619 pacientes submetidos à tomografia computadorizada com emissão de prótons para a predição de risco cardiovascular, as técnicas de ML apresentaram melhores resultados (AUROC 0,81; p < 0.01) do que a análise isolada do exame.^45^

Estudo com mais de 380.000 pacientes do Reino Unido avaliou o uso de técnicas de ML na predição do risco de eventos cardiovasculares em comparação com os algoritmos tradicionais propostos pelo American College of Cardiology e pela American Heart Association^39^. Houve melhoria de até 7,6% na predição de eventos com uso de RNA. Algumas variáveis clínicas que não são valorizadas para doença cardiovascular pelos métodos tradicionais como depressão e uso de corticoides foram importantes para o risco cardiovascular avaliado pelas técnicas de ML.^39^ Este achado foi corroborado por estudo multicêntrico estadunidense em que os parâmetros encontrados para predição de risco cardiovascular diferem daqueles incluídos nas calculadoras de risco tradicionais.^46^

A IA pode contribuir na geração de modelos preditivos mais complexos e específicos para cada indivíduo,^47^ com a incorporação dos componentes genômicos aos escores de risco cardiovascular.^48,49^ A associação dos dados clínicos, sociais, demográficos e genéticos com os exames disponíveis pode permitir uma avaliação mais individualizada, visando à promoção de saúde.^47^

### Diagnóstico

Nos exames cardiológicos, a necessidade de uma equipe médica altamente especializada, a variabilidade de laudos entre os médicos, além do tempo dispensado aos laudos motivaram o estudo das técnicas de ML como ferramenta diagnóstica.^41,50^

Os estudos foram promissores e as modalidades da imagem cardíaca como ecocardiografia, tomografia computadorizada e ressonância nuclear magnética apresentaram boa acurácia em correlacionar alterações estruturais com a etiologia e fisiopatologia de doenças cardiovasculares.^51,52^ Em um estudo com 159 pacientes, o qual utilizou três técnicas de ML para auxiliar na diferenciação ecocardiográfica entre cardiomiopatia hipertrófica e hipertrofia fisiológica de atletas. Os parâmetros encontrados, como: a razão da velocidade transmitral diastólica precoce–tardia (p < 0.01), velocidade diastólica precoce (e’) (p < 0.01) e a análise de *strain* (p < 0.01), foram melhores em sensibilidade e especificidade do que os tradicionalmente usados.^51^

Um algoritmo de ML foi desenvolvido para diferenciar as estenoses coronarianas intermediárias pela angiografia com reserva de fluxo fracionada menor que 0,80 versus maior que 0,80, a partir de dados clínicos e angiográficos. Os resultados foram satisfatórios com acurácia de aproximadamente 80% para predição de reserva de fluxo fracionada menor que 0,8 (AUROC 0,84 a 0,87, IC 95% 0,71 a 0,89). A validação externa do modelo desenvolvido também apresentou resultados similares em 79 pacientes de dois outros centros (AUROC 0,89, IC 95% 0,83 a 0,95).^53^

Em relação à eletrocardiografia, estudos estão sendo desenvolvidos para melhoria dos diagnósticos automáticos.^41^ Através de técnicas de ML, nosso grupo foi capaz de identificar seis classes eletrocardiográficas por meio da análise do ECG de 12 derivações com boa acurácia, comparável ao desempenho que residentes de cardiologia do último ano.^54^ Em pacientes com emergências cardiovasculares hospitalizados, ML teve uma acurácia diagnóstica de cerca de 90% para alterações maiores ao ECG.^54^ Além disso, estudo recente foi capaz de identificar pacientes portadores de fibrilação atrial em ECGs em ritmo sinusal com uma sensibilidade de 79%, especificidade 79,5% e acurácia de 79,4%.^55^

### Limites e Desafios

A utilização das técnicas de ML é crescente, devido ao seu potencial para solucionar problemas nas diversas áreas. Na medicina, os resultados são promissores em diversas especialidades com a expectativa de que a IA possa ser ferramenta de auxílio para a prática clínica.^3,59^ No entanto, ainda é necessária cautela na interpretação e incorporação dos resultados.

Os algoritmos de ML desenvolvidos devem ser reprodutíveis na população geral. Estudos com número pequeno de pacientes, em populações específicas ou com vieses de seleção não permitem a generalização dos seus achados.^60,61^ Ainda que a captação de dados e sua interpretação tenham valor estatístico considerável, os melhores cenários ainda são incapazes de predizer o desfecho em pessoas diferentes.^62^

O erro no processo automatizado pode induzir o profissional a conclusões incorretas, como demonstrado em estudo com 30 residentes em clínica médica que reduziram sua acurácia diagnóstica no laudo de ECG, após a disponibilização de laudos automáticos incorretos.^63^

O avanço da IA na medicina é visto com receio por alguns médicos. A posição alarmista de que ML possa substituir a figura do médico na atenção à saúde tem se mostrado injustificável. Nenhum *software*, até o momento, foi capaz de substituir o aspecto subjetivo da experiência clínica na tomada de decisões favoráveis ao paciente, exatamente, pela medicina não ser uma ciência exata.^64^ A negação ao avanço tecnológico e às ferramentas de IA, hoje disponíveis, tem potencial tão danoso quanto a sua total dependência no atendimento ao paciente. A combinação entre ML e o julgamento clínico tem apresentado melhores resultados em conjunto do que o seu uso isolado.^59^

## Conclusão

O uso de técnicas de ML na medicina deixou o campo teórico e se tornou uma realidade. Embora o uso do ML em medicina ainda esteja em desenvolvimento, estudos mostram a sua aplicabilidade clínica com impacto na avaliação diagnóstica e prognóstica.
