# Supplementary material for: Machine Learning na Medicina: Revisão e Aplicabilidade
Source: Arq Bras Cardiol. 2022 Jan 1;118(1):95–102. [Article in Portuguese] doi: 10.36660/abc.20200596 (PMC8959062; doi:10.36660/abc.20200596)
Supplement: Translation

# Translation: Machine Learning in Medicine: Review and Applicability

**Authors:** Gabriela Miana de Mattos Paixão, Bruno Campos Santos, Rodrigo Martins de Araujo, Manoel Horta Ribeiro, Jermana Lopes de Moraes, Antonio L. Ribeiro

**Affiliations:** 1 Belo Horizonte MG Brazil Universidade Federal de Minas Gerais, Belo Horizonte, MG – Brazil; 2 Sobral CE Brazil Universidade Federal do Ceará, Sobral, CE – Brazil

**Keywords:** Machine Learning, Medicine, Cardiology

## Introduction

Machine learning (ML) is a branch of artificial intelligence (AI) that explores the study and construction of computational algorithms based on data learning,^1,2^ rather than preprogrammed instructions.^3^ The main objective of an ML model is to construct a computer system that learns from a predefined database and, in the end, generates a model for prediction, classification, or detection.

The application of ML in practice is mainly aimed at handling consolidated databases with heterogeneous information where there is a limitation to the use of conventional statistical techniques.^4,5^ ML algorithms are already widespread in different areas, such as banking systems for fraud detection, internet search engines, video surveillance systems, data security, business logistics, robotics, and, in medicine, diagnosis and prognosis.^6^ With the digitalization of medical records, laboratory tests, and imaging, there has been a growth in database are sources for the application of ML techniques, with the aims of prevention, early diagnosis, and treatment of diseases.

This review article provides an introduction to ML structured as follows: definition, learning models, a systematic review of articles on its applicability in medicine, especially cardiology. The objective is to introduce doctors and healthcare professionals to ML as a tool to assist clinical practice.

To structure this review article, the following descriptors in English were searched in the databases PubMed (NCBI) and Medline: “machine learning,” “artificial intelligence,” “unsupervised learning,” “supervised learning,”, “neural networks” and “cardiology.” Prospective and retrospective studies were included, and clinical cases and abstracts presented at conferences (not published as articles) were excluded. Each study's eligibility was assessed by two investigators. Divergent opinions regarding the relevance of articles were addressed by consensus among the authors.

### Machine learning

ML is a subfield of computer science that seeks an intersection between mathematical and statistical techniques and computational algorithms.^3,7^ It uses algorithms with the concept of AI, and it is applied to certain situations to look for patterns within a set of variables in order to predict a specific result of interest.^8,9^

Most of the conventional techniques used in computer systems applied to medicine employ the concept of rule-based algorithms, known as “expert systems.” Thus, developers encode medical knowledge regarding a particular subject for these systems, using rules that are already known. ML techniques, on the other hand, handle a large number of variables, seeking a variety of new combinations that can reliably predict a result, in many cases, in a high volume of data, such as big data.^7^

In 2001, Doug Laney defined the “3 Vs” model to conceptualize the term big data: high volume, high velocity, and high variety of information, which require new processing techniques in order to allow discoveries and optimize processes.^10^ The term big data may refer to either an enormous dataset that no traditional data management tools are able to store or process efficiently or to a type of technology (such as storage facilities, tools, and processes).^11^

The process of developing an ML algorithm is divided into 3 phases: preprocessing, training, and model evaluation (Figure 1). The first phase consists of organizing the databank, defining the research question, and dividing the data into training and testing. During training, learning can take place in a supervised or unsupervised manner.^12–15^ Supervised learning is based on training a data sample where the correct classification has already been assigned, whereas unsupervised learning refers to the capability to learn and organize information when the correct classification has not been assigned.^14^ During the evaluation phase, the model is compared with test data, and the results are generated. Therefore, ML algorithms learn by means of repeated observations, and they establish a mapping pattern in order to label the data and create a model that generalizes the information so that new data (that have never been analyzed by the algorithm) can be accurately and reliably labeled.^15^

**Figure 1:** Phases for developing machine learning algorithms.^15^

It is important to emphasize that the process of developing a ML algorithm must be carried out with a consolidated and validated database, because ML models that are developed with unconsolidated data can generate misleading results.^5^

### Supervised and unsupervised machine learning

The main difference between supervised and unsupervised learning models is in the training algorithm. In unsupervised learning, the ML model extracts the data characteristics and builds a representation without prior knowledge of the labels of each piece of data, that is, it identifies the information classification patterns heuristically. This lack of supervision for the algorithm may be advantageous, because it allows the algorithm to analyze patterns that have not been previously considered.^12–14^

In supervised learning, the ML model possesses knowledge regarding the data labels, that is, the samples are correctly classified. Training is based on the comparison between the result obtained from the model and the previously classified label. This process is repeated until minimum error is reached.^14^

Table 1 summarizes the main characteristics of each type of learning model, as well as their advantages, disadvantages, and practical applicability.

**Table 1:** Comparison between supervised and unsupervised learning processes

	Supervised learning	Unsupervised learning
Definition	Algorithms that learn relationships between input and output attributes based on a set of labeled examples.	Algorithms that attempt to find patterns in data clusters with similar characteristics, looking for unidentified or uninformed categories and outcomes.
Advantages	Analysis of multiple parameters; quick, automatic solution for large-scale questions and high accuracy.	Less human interference in data analysis; excellent for multimodal or multidimensional data sources; allows identification of new outcomes.
Disadvantages	Requires data to be labeled; may be impractical for large volumes of data. Tendency to overfit data.	High cost; complex techniques. It requires a large amount of data to elaborate the algorithm, and it can be challenging to interpret the results.
Main tasks	Regression, classification, prognostic model, and survival analysis.	Reducing dimensionality of the problem and grouping.
Examples of algorithms	Logistic regression, decision trees, random forests, and artificial neural networks.	Principal component analysis, hierarchical clustering, autoencoders, and linear discriminant analysis.

### Machine learning techniques

Several ML techniques have been applied as a form of computer-assisted diagnostic systems, such as artificial neural networks (ANNs), logistic regression, decision tree, random forests, Bayesian network, deep learning, support vector machine (SVM), and others.^16–21^ Some techniques use mathematical models by means of data for learning and/or organization of information.^12^ Others apply mathematical representations with a high degree of abstraction (complex mathematical models). In this case, it is not possible to decipher or interpret the methods used to obtain the prediction, detection, or classification results; these ML models are, thus, known as “black box”.^22^

An ANN is a computational and mathematical model developed to function like the human brain. An ANN possesses several interconnecting elements (predictor layer, hidden layer, and output layer), and the relationship between these layers is inspired by the synaptic connections between neurons (Figure 2).^12,15,23^

**Figure 2:** *Functional structure of an artificial neural network*.^19^

An ANN “learns” by means of these connections between layers (predictors, hidden layer, and results), as well as the weights associated with each layer. Thus, a piece of input data is introduced in the predictor layer and is sent layer by layer. Mathematical processing takes place by sending data from one layer to another, and the weights of these connections are updated according to the error in the result layer, that is, the relationship between the expected result and the obtained result. This process is repeated until the error value is minimal or until a specified interaction value.^12,23,24^

Deep learning differs from more traditional ML techniques to the extent that it processes more robust computational models with multiple processing layers based on ANNs. Thus, the technique of deep learning works in accordance with an ANN, but it possesses a greater number of hidden layers and, consequently, synaptic connections. Each layer reproduces a representation of data from the previous layer, and the learning algorithm can be either supervised or unsupervised.^25,26^

Given the high data volume and complexity involved in working with big data, the autoencoder algorithm is a type of ANN that reduces data dimensionality. In order to do this, the algorithm uses mathematical models with a high degree of abstraction to generate a new dataset with reduced dimensionality and representation as close as possible to the input data. The fundamental difference between an ANN and an autoencoder is that the latter uses unlabeled data during the training phase.^27^

The decision tree algorithm is most used when the dataset is relatively small, and it is developed with a series of yes/no questions to classify data into categories. This algorithm uses a statistical model for data classification or prediction, using the idea of nodes. Each node (question) is divided into possible outcomes, and they branch into other possibilities; this is repeated until the final outcome.^16^ The main advantages of this algorithm are its simplicity and intuitive interpretation.^28^

Random forests are an extension of the decision tree algorithm, and they are widely used to solve classification and regression problems. Decision trees are combined, and each one is trained independently. Its main features are simple theory, fast data analysis, stability in the presence of excessive noise, and an automatic compensation mechanism for biased data samples.^29^

A Bayesian network is another technique that is widely applied to medicine. It consists of Bayesian statistical methods based on the theoretical foundation that consistent subjective beliefs of specialists in a given area can be expressed in a probabilistic structure.^17^

SVM is a supervised ML method that is widely used in bioinformatics. This algorithm uses the idea of error minimization, working with the statistical theory of learning and optimization. In addition to binary classification, SVM can be used in continuous data regression, called support vector regression. The results obtained with the use of SVM are comparable to those of ANNs, presenting an easy training process and working with high data dimensionality. It, therefore, reaches a compromise between less complexity and error.^30,31^

In this manner, each algorithm applies different techniques regarding how to learn from observations and how to carry out mapping of a set of predictors for the final result. It must generalize information so that a task can be performed correctly with new inputs that have not been previously analyzed by the model.^14^

### Machine learning in medicine

Since the past century, researchers have been exploring different applications of ML techniques in all fields of medicine.^32^ Medical research involving ML has grown exponentially over the past few decades. Data from PubMed (NCBI) and Medline, involving the descriptors “machine learning,” “artificial intelligence,” “unsupervised learning,” “supervised learning,” and “neural networks,” revealed 113,127 articles published between 1951 and 2019 (Figure 3). When the descriptor “cardiology” was added as a mandatory condition in the search for the other terms, 888 studies were found, with distribution similar to the previous one, between the years 1986 and 2019.

**Figure 3:** *Number of articles per year and cumulative during the period from 1951 to 2019 in PubMed and Medline*.

The capability of ML algorithms to recognize patterns and predict diagnoses has been widely applied to different areas of healthcare.^33–36^ In dermatology, an ANN was able to differentiate dermatological lesions as benign versus malignant, based on more than 129,000 cases, with results similar to those of a committee of 21 dermatologists.^35^ In the field of psychiatry, a study with ML techniques reduced the number of diagnostic criteria from 29 to 8, with 100% accuracy in 612 patients with confirmed diagnosis of autistic spectrum disorder.^36^

The addition of mobile technologies, such as smartphones and smartwatches, applied to the area of healthcare has added another dimension to ML, making it possible to read large quantities of personal data in learning algorithms.^37^ Within feedback systems, mobile technology is able to be a biometric device (for example, measuring blood glucose levels), capable of targeting real-time clinical interventions, based on algorithms that continuously update patients’ personal information.^38^ Technology is able to simplify diagnostic processes and facilitate clinical practice.

### Machine learning in cardiology

Advances in computational capacity in recent decades have especially impacted the field of detection and prediction of cardiovascular diseases through interpretation of data, such as studies of medical records; imaging exams; and biological, genomic, and molecular evaluation databanks.^32^ Cardiology is one of the areas with the greatest impact on scientific production using ML techniques (Table 2). From the prediction of cardiovascular events^39^ to the improvement of electrocardiographic and imaging diagnoses,^40,41^ AI has been an important tool for scientific research.

**Table 2:** Articles on the use of machine learning in cardiology

Article	Main results
Can machine-learning improve cardiovascular risk prediction using routine clinical data?^38^	The algorithm was able to predict 4998 of 7404 positive cases (sensitivity 67.5%, PPV 18.4%) and 53,458 of 75,585 negative cases (specificity 70.7% and NPV 95.7%), with a gain of 355 patients (+7.6%) who developed cardiovascular diseases, compared to the traditional method.
Deep neural networks can predict mortality from 12-lead electrocardiogram voltage data^43^	By means of isolated analysis of ECG using a ML algorithm, it was possible to predict 1-year all-cause mortality with AUC = 0.84 and p < 0.05.
Phenomapping for the Identification of Hypertensive Patients with the Myocardial Substrate for Heart Failure with Preserved Ejection Fraction^56^	A group of 1273 patients with hypertension was evaluated using ML techniques, using clinical, laboratory, and echocardiography data. It was possible to identify a group of patients at a higher risk of developing heart failure with preserved ejection fraction who were likely to benefit from more intensive medical treatment.
Cognitive Machine-Learning Algorithm for Cardiac Imaging: A Pilot Study for Differentiating Constrictive Pericarditis From Restrictive Cardiomyopathy^57^	They used ML techniques to differentiate constrictive pericarditis from restrictive cardiomyopathy with a ROC curve of 96.2% and accuracy greater than 90%.
Structured learning algorithm for detection of nonobstructive and obstructive coronary plaque lesions from computed tomography angiography^58^	The ML algorithm was able to detect coronary lesions greater than or equal to 25% with 93% sensitivity, 95% specificity, and 94% accuracy in 42 coronary angiographies.
A deep neural network learning algorithm outperforms a conventional algorithm for emergency department electrocardiogram interpretation^54^	Automatic analysis using the ML method for reading ECG in an emergency department obtained sensitivity (88.7% versus 92.0%, p < 0.086), specificity (94% versus 84.7%, p < 0.0001), PPV (88.2% versus 75.4%, p < 0.0001), and accuracy (92.2% versus 87.2%, p < 0.0001), compared to the conventional automatic method.
Automatic Diagnosis of the Short-Duration 12-Lead ECG using a Deep Neural Network: the CODE Study^53^	A trained neural network was able to detect 6 classes of electrocardiographic abnormalities with specificity greater than 99% and performance greater than 80%, compared to last-year cardiology residents.
An artificial intelligence-enabled ECG algorithm for the identification of patients with atrial fibrillation during sinus rhythm: a retrospective analysis of outcome prediction^55^	ML software was able to detect patients with atrial fibrillation, based on ECG in sinus rhythm, with a sensitivity of 79%, specificity of 79.5%, and accuracy of 79.4%.

*ECG: electrocardiogram; ML: machine learning; NPV: negative predictive value; PPV: positive predictive value.*

### Prognosis

Several cardiovascular risk scores have been developed in order to predict cardiovascular events and identify individuals with higher cardiac risks, for primary prevention.^42^ However, in spite of all the advances in diagnostic workup and therapy in cardiology, there is still a population at risk that is has not been identified by traditional methods.^43^ It is desirable to recognize potential non-traditional risk factors, and the use of new technologies, such as AI, has become a promising method in this search.

The prediction of all-cause mortality over a 1-year period, based on isolated analysis of electrocardiogram (ECG) has shown promising results (AUROC 0.87; p < 0.05).^44^ It is interesting to underscore that a blind analysis of the same ECGs by 3 cardiologists suggested that the patterns which ML found to predict mortality were not apparently visible on conventional medical assessment.^44^

In a study including 2619 patients who underwent computerized tomography with proton emission for prediction of cardiovascular risk, ML techniques showed better results (AUROC 0.81; p < 0.01) than isolated analysis of the exam.^45^

A study with more than 380,000 patients in the United Kingdom evaluated the use of ML techniques to predict the risk of cardiovascular events in comparison with the traditional algorithms proposed by the American College of Cardiology and the American Heart Association.^39^ There was an improvement of up to 7.6% in the prediction of events with the use of ANN. Some clinical variables that are not valued for cardiovascular disease by traditional methods, such as depression and corticosteroid use, were important to cardiovascular risk assessed by ML techniques^39^. This finding was corroborated by a multicenter study from the United States where the parameters found for cardiovascular risk prediction differed from those included in traditional risk calculators.^46^

AI can contribute to the generation of more complex and specific predictive models for each individual,^47^ by incorporating genomic components in cardiovascular risk scores.^48,49^ The association of clinical, social, demographic, and genetic data with the available exams can allow more individualized assessment, with the aim of health promotion.^47^

### Diagnosis

In cardiac exams, the need for a highly specialized medical team a variability of reports among physicians, and time spent on reports have led to the study of ML techniques as a diagnostic tool.^41,50^

The studies have been promising, and cardiac imaging modalities such as echocardiography, computed tomography, and nuclear magnetic resonance have shown good accuracy in correlating structural changes with the etiology and pathophysiology of cardiovascular diseases.^51,52^ In a study with 159 patients, 3 ML techniques were used to aid in the echocardiographic differentiation between hypertrophic cardiomyopathy and physiological hypertrophy in athletes. The parameters found, such as early-to-late transmitral diastolic velocity ratio (p < 0.01), early diastolic velocity (e’) (p < 0.01), and strain analysis (p < 0.01), were better in sensitivity and specificity than those that are traditionally used.^51^

A ML algorithm was developed to differentiate intermediate coronary stenosis on angiography with fractional flow reserve less than 0.80 versus greater than 0.80, based on clinical and angiography data. The results were satisfactory, with an accuracy of approximately 80% for prediction of fractional flow reserve less than 0.8 (AUROC 0.84 to 0.87, 95% CI 0.71 to 0.89). The external validation of the developed model also showed similar results in 79 patients from 2 other centers (AUROC 0.89, 95% CI 0.83 to 0.95).^53^

In relation to ECG, studies are being developed to improve automatic diagnoses.^41^ By means of ML techniques, our group has been able to identify 6 ECG classes through 12-lead ECG analysis, with good accuracy, comparable to the performance of last-year cardiology residents.^54^ In hospitalized patients with cardiovascular emergencies, ML had diagnostic accuracy of about 90% for major ECG changes.^54^ Furthermore, a recent study was able to identify patients with atrial fibrillation on ECG in sinus rhythm with a sensitivity of 79%, specificity of 79.5%, and accuracy of 79.4%.^55^

### Limits and challenges

The use of ML techniques is growing, due to their potential to solve problems in different areas. In medicine, the results have been promising in several specialties, with the expectation that AI can be a tool to assist clinical practice.^3,59^ Nevertheless, it is still necessary to be cautious when interpreting and incorporating the results.

The ML algorithms developed must be reproducible in the general population. Studies with small numbers of patients, in specific populations, or with selection biases do not allow for generalization of their findings.^60,61^ Even though data capture and interpretation have considerable statistical value, the best scenarios are still not capable of predicting the outcome in different people.^62^

An error in an automated process can lead professionals to incorrect conclusions, as demonstrated in a study with 30 internal medicine residents whose diagnostic accuracy for ECG reports was reduced when they were provided with incorrect automatic reports.^63^

Some doctors have viewed the advancement of AI in medicine with concern. The alarmist position that ML might replace doctors in healthcare has proved to be unjustified. No software, so far, has been able to replace the subjective aspect of clinical experience in making favorable decisions for the patient, precisely because medicine is not an exact science.^64^ The denial of technological advancement and the AI tools that are available today is as potentially damaging as total dependence on ML for patient care. The combination of ML and clinical judgment has shown better results together than its isolated application.^59^

## Conclusion

The use of ML techniques in medicine has left the field of theory and gone on to become a reality. Although the use of ML in medicine is still in development, studies have demonstrated its clinical applicability, with an impact on diagnostic and prognostic evaluation.
